# Knowledge, attitudes and practices about malaria among communities: Comparing epidemic and non-epidemic prone communities of Muleba district, North-western Tanzania

**DOI:** 10.1186/1471-2458-10-395

**Published:** 2010-07-05

**Authors:** Safari M Kinung'hi, Fabian Mashauri, Joseph R Mwanga, Soori E Nnko, Godfrey M Kaatano, Robert Malima, Coleman Kishamawe, Stephen Magesa, Leonard EG Mboera

**Affiliations:** 1 National Institute for Medical Research, Mwanza Centre, P. O. Box 1462, Mwanza, Tanzania; 2 National Institute for Medical Research, Amani Centre, P. O. Box 81, Muheza, Tanzania; 3 National Institute for Medical Research, Headquarters, P.O. Box 9653, Dar-Es-Salaam, Tanzania

## Abstract

**Background:**

Muleba district in North-western Tanzania has experienced malaria epidemics in recent years. Community knowledge, attitudes and practices are important in enhancing disease control interventions. This study investigated determinants of malaria epidemics in the study area in relation to household knowledge, attitudes and practice on malaria.

**Methods:**

A community based cross-sectional survey involving 504 study participants was conducted between April and June 2007 using a structured questionnaire focusing on knowledge, attitudes and practices of community members in epidemic and non-epidemic villages about malaria transmission, signs and symptoms, treatment, prevention and control. Multivariate logistic regression analysis was used to assess determinants of malaria epidemics.

**Results:**

A total of 504 respondents (males = 36.9%) were interviewed. Overall, 453 (90.1%) mentioned malaria as the most important disease in the area. Four hundred and sixty four respondents (92.1%) knew that malaria is transmitted through mosquito bite. A total of 436 (86.7%), 306 (60.8%) and 162 (32.1%) mentioned fever, vomiting and loss of appetite as major symptoms/signs of malaria, respectively. Of those interviewed 328 (65.1%) remembered the recent outbreak of 2006. Of the 504 respondents interviewed, 296 (58.7%) reported that their households owned at least one mosquito net. Three hundred and ninety seven respondents (78.8%) knew insecticides used to impregnate bed nets. About two thirds (63.3%) of the respondents had at least a household member who suffered from malaria during the recent epidemic. During the 2006 outbreak, 278 people (87.2%) sought treatment from health facilities while 27 (8.5%) obtained drugs from drug shops and 10 (3.1%) used local herbs. Logistic regression analysis showed that household location and level of knowledge of cause of malaria were significant predictors of a household being affected by epidemic.

**Conclusions:**

Residents of Muleba district have high level of knowledge on malaria. However, this knowledge has not been fully translated into appropriate use of available malaria interventions. Our findings suggest that household location, ineffective usage of insecticide treated nets and knowledge gaps on malaria transmission, signs and symptoms, prevention and control predisposed communities in the district to malaria epidemics. It is important that health education packages are developed to address the identified knowledge gaps.

## Background

Malaria is the leading public health problem in Tanzania. The disease contributes between 39.4% and 48% of all outpatient children under the age of five years and among those aged five years and above, respectively [1]. Malaria is the major cause of hospital admissions accounting for 33.4% in children under the age of five years and 42.1% in those aged five years and above. The disease is responsible for more than one-third of deaths in children under the age of five years and up to one-fifth of deaths among pregnant women [1]. Malaria occurs in all parts of Tanzania with varying levels of endemicity ranging from unstable seasonal malaria, stable malaria with seasonal variations to stable perennial malaria [2,3]. Unstable seasonal malaria occurs in areas with low malaria transmission of not more than three months a year. Such areas include the northern and southern highlands and arid areas of central Tanzania with altitude up to 2000 m above sea level, temperature between 20°C and mean vapour pressure of 13-15 millibars. In such areas, malaria may occur as epidemics normally associated with increased morbidity and mortality. About 25% of the 38.7 million Tanzanians live in malaria unstable epidemic prone areas [4].

During the past six decades, malaria epidemics have been reported in some districts of Tanzania. These include the semi-arid areas of Dodoma and the East and West Usambara Mountains of Muheza and Lushoto; Babati, Hanang, Mbulu, Muleba and Loliondo Districts [2,5]. Many of the observed malaria epidemics were caused by an increase in immigrants into and from malarious areas, ecological changes, poor surveillance system and degradation of healthcare infrastructures [2,6,7]. A malaria epidemic is defined as an abrupt increase in malaria transmission that exceeds by far the inter-seasonal variation normally experienced in a given area and often associated with increased morbidity and mortality [8]. This occurs when the equilibrium between the human host population, malaria parasites and the malaria vector population is disturbed [9]. The conceptual framework for increased malaria transmission in Sub-Saharan Africa was discussed by Robert *et al *[10] and Deressa *et al *[11]. Briefly, malaria epidemics are linked to environmental changes and increased mean rainfall and ambient temperatures, changes in land use patterns, malaria vector dynamics, host immune status and individual or community factors such as socio-economic status, population movement, knowledge on malaria and protective behaviours [7,10-13]. Malaria epidemics can also be caused by breakdown of malaria intervention programs and drug or insecticide resistance [12]. Periods of drought are a common characteristic prior to most malaria epidemics [6,7]. Such links between climate variability, vector dynamics, droughts, food shortage and epidemics have been reported during the devastating malaria epidemics in Tanzania [6,7,14-18].

Muleba district is known to be a malaria epidemic prone area with unstable transmission of varying seasonality. The highest peak of malaria transmission is usually reached between May - July and November-January, which results from proceeding rain seasons. In 1997, a widespread malaria epidemic was reported affecting parts of North-west Tanzania including Kigoma and Kagera regions [7,19]. Poor rainfall in the previous wet season, associated with low food production and poor economic situations, were responsible for the epidemic. The 1997/1998 epidemic in Muleba was also associated with unusually heavy El Nino-Southern Oscillation (ENSO) rains, lack of antimalarial drugs and ineffective chloroquine [20]. An assessment carried out in Muleba by Garay [7] indicated that malaria admission and malaria specific mortality for January-March 1998 had risen four and 12 fold respectively, compared to the previous year's figure. In May 2006, the Muleba district authority noticed a drastic increase in number of outpatient and inpatient malaria cases accompanied by increased mortality especially of underfive children. There was a two-fold increase in the number of outpatient malaria cases from 2573 in January 2006 to 4388 in May 2006 in underfive children. There was also an increase of inpatient underfive children from 1094 (January 2006) to 1927 (May 2006). The Case Fatality Rate (CFR) for underfives increased from 10/1000 (January 2006) to 29/1000 (May 2006) (Ministry of Health, unpubl).

Although many studies in Tanzania and other African countries have linked socio-economic and behavioural factors, community knowledge, attitudes and practices with malaria [21-27], no studies which have established such a link between these factors and malaria epidemics. An understanding of knowledge, attitudes and practices among communities in epidemic and non-epidemic areas and identification of the main factors that influenced malaria treatment and protective behaviours during epidemics is therefore important in the design and implementation of appropriate malaria epidemic control strategies. The current study investigated household's knowledge, attitudes and practices concerning malaria epidemics in Muleba district and explored determinant factors that might have contributed to malaria epidemics in the district.

## Methods

### Description of the study area

Muleba District (1°45'N, 31°40'E) is in the North-western part of Tanzania with an area of 10,739 km^2^, of which 62.0% consists of Lake Victoria. Most parts of the district lie at 1200-1500 m above sea level. Administratively the district has 5 divisions, 31 wards, and 134 villages. It has a population of 425,172 people [28] with 85,035 (20%) being children under the age of five years. The district has 36 health facilities, 3 of them being hospitals (Rubya, Kagondo and Ndolage). Others are health centres (4) and dispensaries (29). The district has two rain seasons which occur in March - June and September-December during which malaria transmission peaks.

### Study design and sampling procedures

The study was a community based cross-sectional survey conducted between April and June, 2007 in six selected villages. Study villages were selected using a multistage simple random sampling procedure and a cluster sampling procedure as the final stage. Selection was made with the assistance of village and sub village heads. In the first stage, names of villages with the history of been affected by the epidemic of 2006 were listed from records obtained from the district medical officer's office. From this list three villages namely, Ijumbi (31°6'E; 01°7), Nshambya (31°6' E; 01°7' S) and Ikondo (31°6' E; 01°8') were randomly selected. Another three villages namely Bushemba (31°6'; 01°7'), Kibanga (31°6'E; 01°9') and Bunywambele (31°. 6 E'; S. 01°. 6') were selected randomly from a list of villages not affected by the epidemic and were used as control villages. In the second stage, for each selected village, a list of all sub villages was made from which two sub villages were randomly selected making a total of 12 sub villages (six sub villages in the epidemic area and six sub villages in the non-epidemic area). In the third stage, with the assistance of sub village heads, a list of all households with at least 5 household members was made from which 16 to 20 households were randomly selected. It was estimated that 16 to 20 households per sub village each with al least 5 household members would give an overall sample size of 400 people aged 15 years and above which was considered sufficient for the study. In the fourth (final) stage, all household members in the selected households aged 15 years and above were selected and invited for interview. In total 504 participants aged 15 years and above attended the interviews and were included in the study.

### Data collection methods

A structured, pre-tested questionnaire focusing on community knowledge, attitudes and practices on malaria and malaria epidemics was administered to the 504 eligible participants. The questionnaire examined knowledge of participants about malaria transmission, signs and symptoms as well as treatment, prevention and control. Household treatment seeking and preventive behaviour, mosquito nets usage and coverage was explored and documented. In addition, socio-economic factors which could predispose communities to malaria epidemics were investigated.

### Data management and analysis

In the field, data was collected in standardized questionnaire and data collection forms and checked for errors and completeness. Data was then counterchecked before entry into DbaseV (Borland International, Scotts Valley, California, USA) using the double entry system. Summary statistics was performed using STATA version 10 (STATA Corp., Texas, USA). Comparison of proportions between epidemic and non-epidemic villages was performed by cross-tabulation using the Chi-square test. The fisher's exact test was used where the figures in cells were less than 5. Logistic regression analysis was performed to assess factors which could have predisposed study communities to the malaria epidemic of 2006. Independent variables included in the model were household location, education level of household head, number of people in the household, knowledge of malaria, ownership and use of mosquito nets, knowledge and use of insecticides to protect against malaria, ownership and use of antimalarials for home management of malaria and type of antimalarials used. A p-value of less than 0.05 was considered significant.

### Ethical considerations

Ethical clearance certificate (NIMR/HQ/R.8a/Vol. IX/503) to conduct the study was granted by the Medical Research Coordination Committee (MRCC) of the National Institute for Medical Research (NIMR), Tanzania that acts as the national ethics review board in Tanzania. Before commencement of the study, the principal investigator and his research team conducted meetings with local leaders and communities in all selected villages during which the objectives of the study including procedures to be followed were explained. Participants who consented to participate in the study were invited to attend interviews at selected central places in the villages such as schools. Study identification numbers were used instead of participant names and information collected was kept confidential. Feedback to the study population was conducted in the form of dissemination meetings after completion of the study.

## Results

### Knowledge about causes, signs and symptoms of malaria and malaria epidemics

A total of 504 participants were interviewed using a general questionnaire of whom 186 (36.9%) were males and 318 (63.1%) were females. The socio-demographic characteristics of the study population are shown in table 1. Overall, 453 participants (90.1%) mentioned malaria as the most important disease followed by HIV/AIDS (50.2%). Other important health problems mentioned included schistosomiasis (17%), respiratory infections including tuberculosis (13.1%) and malnutrition (5.6%). Of the 504 respondents, 449 (89.3%) mentioned malaria as the first cause of death while another 226 (50.6%) and 48 (18.1%) mentioned HIV/AIDS and diarrhoeal diseases as second and third causes of death, respectively.

**Table 1:** Socio-demographic characteristics of the study population in Muleba district (n = 504)

Variable	Number of respondents (%)			P-Value
		
	Overall	Epidemic area	Non-Epidemic area	
Age group				
15-24	102 (20.3)	62 (24.5)	40 (10.0)	< 0.001
25-34	119 (23.7)	51 (20.2)	68 (27.2)	0.065
35-44	143 (28.4)	74 (29.3)	69 (27.6)	0.673
45-54	75 (14.9)	39 (15.4)	36 (14.4)	0.753
55-64	31 (6.2)	18 (7.1)	13 (5.2)	0.375
65+	33 (6.6)	9 (3.6)	24 (9.6)	< 0.01
Education of respondents				
None	77 (23.6)	55 (24.4)	22 (21.6)	0.580
Primary school	237 (72.5)	161 (71.6)	76 (74.5)	0.586
Secondary school	13 (4.0)	9 (4.0)	4 (3.9)	0.966
Education of household head				
None	14 (9.9)	3 (4.8)	11 (13.7)	0.077
Primary	116 (81.7)	50 (80.7)	66 (82.5)	0.783
Secondary	8 (5.6)	6 (9.7)	2 (2.5)	0.065
College	4 (2.8)	3 (4.8)	1 (1.3)	0.212
Marital status				
Single	75 (14.9)	49 (19.3)	26 (10.4)	< 0.01
Married	376 (74.6)	191 (75.2)	185 (74.0)	0.757
Divorced	9 (1.8)	3 (1.2)	6 (2.4)	0.314
Separated	4 (0.8)	2 (0.8)	2 (0.8)	1.000
Widowed	40 (7.9)	9 (3.5)	31 (12.4)	< 0.001
Marriage type				
Monogamy	318 (85.0)	232 (88.6)	86 (76.8)	< 0.01
Polygamy	56 (15.0)	30 (11.5)	26 (23.2)	< 0.01

Majority of respondents (92.1%) associated malaria transmission with mosquito bites with no significant differences in responses between epidemic and non-epidemic villages (p = 0.349). Some respondents mentioned bedbugs (0.6%), ticks (1.6%), contaminated water (15%), basking in sun-shine (2.6%), working in the rains (1%) and witchcraft (0.4%). Fever was the most frequently mentioned symptom of malaria. Very few respondents identified convulsions, anaemia and jaundice as signs for malaria (Table 2). Out of total respondents, 328 (65.1%) remembered the malaria epidemic of 2006. The outbreak was mainly associated with natural causes including heavy rains (Table 3). A significantly larger proportion of respondents from non-epidemic villages 119 (64.3%) than epidemic villages 83 (41.1%) associated malaria epidemics with natural causes such as heavy rains (p < 0.01). Other factors which were listed to have caused the epidemic included drought, food shortage, poor environmental sanitation and not using mosquito nets.

**Table 2:** Signs and symptoms for malaria in children under five years in Muleba district (n = 504)

Sign/Symptom	Number of respondents (%)			P-Value
		
	Overall	Epidemic area	Non-Epidemic area	
Fever				
Yes	436 (86.5)	213 (83.9)	223 (89.6)	
No	67 (13.3)	41 (16.1)	26 (10.4)	0.060
Vomiting				
Yes	306 (60.8)	149 (58.9)	157 (62.8)	
No	197 (39.2)	104 (41.1)	93 (37.2)	0.370
Convulsions				
Yes	144 (28.6)	75 (29.5)	69 (27.6)	
No	360 (71.4)	179 (70.5)	181 (72.4)	0.637
Loss of consciousness				
Yes	15 (3)	15 (4.3)	0	
No	489 (97)	334 (97.5)	155 (100.0)	< 0.01
Loss of appetite/refuse feeding				
Yes	162 (32.1)	91 (35.8)	71 (28.4)	
No	342 (67.9)	163 (64.2)	179 (71.6)	0.075
Anaemia				
Yes	66 (13.1)	31 (12.3)	35 (14.0)	
No	437 (86.9)	222 (87.8)	215 (86.0)	0.572
Jaundice				
Yes	58 (11.5)	19 (7.5)	39 (15.6)	
No	446 (88.5)	235 (92.52)	211 (84.4)	0.004

**Table 3:** Perceived causes and impact of the most recent malaria epidemic in Muleba district (n = 387)

Variable	Number of respondents (%)			P - Value
		
	Overall	Epidemic area	Non-Epidemic area	
**Causes of the epidemic**				
Natural cause (heavy rains)				
Yes	202 (52.2)	83 (41.1)	119 (64.3)	
No	185 (47.8)	119 (58.9)	66 (35.7)	< 0.01
Changes in farming system				
Yes	15 (3.9)	9 (4.4)	6 (3.2)	
No	372 (96.1)	193 (95.5)	179 (96.7)	0.539
Poor health services				
Yes	34 (8.8)	20 (9.9)	14 (7.6)	
No	353 (91.2)	182 (90.1)	171 (92.4)	0.425
**Impact of the epidemic**				
Many people died				
Yes	368 (95.1)	189 (93.1)	179 (97.3)	
No	19 (4.9)	14 (6.9)	5 (2.7)	0.056
Many people had malaria				
Yes	113 (29.2)	64 (31.5)	49 (26.6)	
No	274 (70.8)	139 (68.5)	135 (73.4)	0.290
Many people were hospitalized				
Yes	106 (27.4)	52 (25.6)	54 (29.4)	
No	281 (72.6)	151 (74.4)	130 (70.1)	0.402

### Health seeking and treatment during malaria epidemics

Out of 504 respondents, 319 (63.3%) had at least one family member who suffered from malaria during the epidemic of 2006. Children under the age of 5 years were the most affected (53% of responses), followed by those aged between 5 - 19 years (Table 4). Sulfadoxine-pyrimethamine was frequently mentioned as the drug used to treat malaria among respondents with no significant differences in responses between epidemic and non-epidemic villages (p = 0.063) (table 4). Out of those interviewed, only 85 (17%) reported to keep antimalarials in their homes during the time of the epidemic. During the time of the study (April to June, 2007), artemisinin based combination therapy (ACT) particularly artemether-lumefantrine (ALU) was the first line antimalarial drug in Tanzania [29,30]. The decision to change from sulphadoxine-Pyrimethamine (SP) to ACT in Tanzania was made in 2005 but implementation was officially made in 2006 [29].

**Table 4:** Susceptibility patterns with respect to age, health seeking and treatment during the malaria epidemic of 2006 in Muleba district, Tanzania (n = 319)

Variable	Number of respondents (%)			P-Value
		
	Overall	Epidemic area	Non-Epidemic area	
**Susceptibility by age group (years)**				
Below 5	169 (53)	116 (50.0)	53 (60.9)	0.082
5-19	98 (30.7)	75 (32.3)	23 (26.4)	0.308
20-35	14 (4.4)	11 (4.5)	3 (3.5)	0.692
36-65	30 (9.4)	25 (10.8)	5 (5.8)	0.173
Above 65	8 (2.5)	5 (2.2)	3 (3.5)	0.512
**Source of healthcare services**				
Health facility	278 (87.2)	204 (87.9)	74 (85.1)	0.506
Drug shop	27 (8.5)	19 (8.2)	8 (9.2)	0.775
Self treatment using herbs	10 (3.1)	7 (3.0)	3 (3.5)	0.820
**Drug used for treatment of malaria**				
Sulfadoxine-pyrimethamine				
Yes	92 (29)	74 (31.9)	18 (21.2)	
No	225 (71)	158 (68.1)	67 (78.8)	0.063
Amodiaquine				
Yes	63 (20)	42 (18.1)	21 (24.7)	
No	254 (80.1)	190 (81.9)	64 (75.3)	0.192
Antermisinin compounds				
Yes	24 (7.6)	16 (6.9)	8 (9.4)	
No	293 (92.4)	216 (93.1)	77 (90.6)	0.456
Quinine				
Yes	78 (24.6)	25 (25.4)	19 (22.4)	
No	239 (75.4)	173 (74.6)	66 (77.7)	0.591
Chloroquine				
Yes	15 (4.7)	11 (4.7)	4 (4.7)	
No	302 (95.3)	221 (95.3)	81 (95.3)	1.000
Don't know				
Yes	63 (79.8)	38 (84.4)	25 (73.5)	
No	16 (20.3)	7 (15.6)	9 (26.5)	0.236

### Knowledge about malaria prevention and control methods

Of the 504 respondents interviewed, 491 (97.4%) knew mosquito nets. A total of 397 respondents (78.8%) knew insecticides used to impregnate nets. More respondents from epidemic (86.5%) than from non-epidemic villages (71.3%) were knowledgeable on the types of insecticides used in malaria control (P = 0.030) (Table 5). Other types of repellants used to prevent mosquito bites included burning of herbs locally known as *ebishambo*, eucalyptus leaves and neem leaves. Despite knowledge and availability of mosquito nets in the area, a significant proportion of respondents 215 (45.6%) said that mosquito nets were not affordable to most people. The price of a medium sized mosquito net during the time of the study was between US$1.6 - 3.0. The affordable price according to most respondents (72%) was US$ 1.6 or less.

**Table 5:** Insecticides used to protect against mosquitoes in Muleba district (n = 395)

Type of insecticide	Number of respondents (%)			P-Value
		
	Overall	Epidemic	Non-Epidemic	
Permethrin (NGAO^®^)				
Yes	313 (79.3)	179 (86.5)	134 (71.3)	
No	82 (20.8)	28 (13.5)	54 (28.7)	0.030
Mosquito coils				
Yes	35 (8.9)	17 (8.2)	18 (9.6)	
No	360 (91.1)	190 (91.8)	170 (90.4)	0.625
Aerosal sprays				
Yes	23 (5.8)	16 (5.8)	7 (5.8)	
No	372 (94.2)	259 (94.2)	113 (94.2)	1.000

Out of the 504 people interviewed, 296 (58.7%) said their households owned at least one mosquito net (table 6). The number of households owning at least one mosquito net was significantly higher (p = 0.019) in non-epidemic villages compared to epidemic villages. Most of the respondents (72.6%) bought their nets while the rest acquired them for free. Those who acquired their nets for free obtained them from faith based organizations (24.6%) and as gifts from relatives (2.7%). Respondents also mentioned clearing bushes around houses, cleaning house environment, destroying mosquito breeding grounds and closing doors and windows early after sunset as self protection measures against mosquitoes. Despite good knowledge and usage of mosquito nets, 30 respondents (10.7%) said that insecticide treated nets (ITNs) had harmful effects to the health of users. Sixty-eight (7.5%) respondents said ITNs were harmful to pregnant women and 6 respondents (2.6%) said ITNs were harmful to young children.

**Table 6:** Mosquito net and insecticides usage in Muleba district, Tanzania (n = 294)

Variable	Number of respondents (%)			P-Value
		
	Overall	Epidemic area	Non-Epidemic area	
Does your household own mosquito net/nets?				
Yes	296 (58.7)	193 (55.3)	103 (66.4)	
No	208 (41.3)	156 (44.7)	52 (33.6)	0.019
Who sleeps under a net?				
All people	132 (45.4)	89 (47.1)	43 (42.2)	0.404
Father and mother only	14 (4.8)	10 (5.3)	4 (3.9)	0.578
Father, mother and young children	44 (15.1)	30 (15.9)	14 (13.7)	0.602
Young children only	37 (12.7)	25 (13.2)	12 (11.8)	0.721
Mother and young children only	23 (7.9)	15 (7.9)	8 (7.8)	0.975
Other	41 (14.1)	20 (10.59)	25 (24.5)	< 0.01
Season when mosquito nets are used				
All year round	206 (70)	137 (71.7)	69 (67.0)	0.401
Rain season only	70 (23.8)	42 (22.0)	28 (27.2)	0.318
Other	18 (6.1)	12 (6.3)	6 (5.8)	0.865
Net re-treatment is done				
Yes	2 (1.2)	2 (1.7)	0	-
No	161 (98.2)	114 (97.4)	47 (100.0)	0.265
Don't know	1 (0.6)	1 (0.9)	0	-
Insecticide for net treatment available in the household				
Yes	89 (17.8)	64 (18.6)	25 (16.1)	0.499
No	408 (81.6)	279 (80.9)	129 (83.2)	0.539
Don't know	3 (0.6)	2 (0.6)	1 (0.7)	0.670
Other means of self protection against mosquitoes				
Insecticide sprays	9 (1.8)	4 (1.6)	5 (2.0)	0.867
Insecticide coils	33 (6.6)	19 (7.6)	14 (5.6)	0.658
Burn local herbs	53 (10.7)	30 (12.0)	23 (9.3)	0.630
Mosquito repellents	28 (5.7)	13 (5.2)	15 (6.1)	0.829

### Determinants of malaria epidemics

Logistic regression analysis was performed to identify factors which were associated with the risk of a household having at least one family member who suffered from malaria during the epidemic of 2006. In the bivariate analysis, factors which were independently associated with the risk of a household being affected by the epidemic were household location and knowledge of cause of malaria. Households located in the non-epidemic area had a reduced risk [OR 0.66 (95% CI = 0.46 - 0.94), p = 0.02] of being affected by the epidemic compared to household in the epidemic area. Knowledge of cause of malaria was associated with protection from the epidemic [OR 0.40 (95% CI 0.21 - 0.76), p = 0.006]. Households not using a mosquito net during the epidemic and those with more than 5 household members had an increased risk of being affected by the epidemic but these associations were not significant (p > 0.05). Likewise, households with members not using mosquito nets throughout the year had an increased risk of being affected by the epidemic but the association was not significant (p = 0.164). In the multivariate logistic regression model, factors that remained significantly associated with the risk of a household being affected by the epidemic were household location and knowledge of cause of malaria (table 7).

**Table 7:** Multivariate logistic regression analysis showing predictors of a household being affected by malaria epidemic in Muleba district (n = 294)

Independent variable	Categories	Adjusted Odds Ratio (95% CI)	P-Value
Household location	Epidemic area	1	
	Non-Epidemic area	0.48 (0.29 - 0.79)	0.004
Knowledge of cause of malaria	No	1	
	Yes	0.38 (0.15 - 0.98)	0.045
Use of mosquito nets in relation to season	Throughout the year	1	
	Rain season only	1.60 (0.90 - 2.90)	0.123
	Other seasons	1.16 (0.40 - 3.30)	0.780

## Discussion

Most studies which have attempted to investigate determinants of malaria epidemics in East Africa have used quantitative epidemiological methods focusing on biomedical factors. This study is unique in that it has approached the subject by focusing on socio-economic, knowledge, attitudes, practices and other behavioural factors. Findings of this study indicate that malaria is an important public health problem in Muleba and that community knowledge about its transmission, signs and symptoms, treatment and prevention is high. Furthermore, respondents had good knowledge of the cause, impact and prevention strategies against malaria epidemics. Majority of respondents reported health facilities as the right place where to seek health care for malaria treatment. The high level of knowledge, though comparable to what has been reported by other studies could be attributed in part to health education campaigns that followed immediately after the epidemic. As regards health seeking, treatment and prevention behaviour, the observations of this study is consistent with findings of other studies [31-35] that demonstrated that people with good knowledge about malaria cause and transmission do take appropriate treatment and preventive measures. However during the epidemic, majority of respondents used SP and other monotherapy antimalarials for treatment of malaria cases instead of ACTs which was the first line antimalarial drug according to national malaria treatment guideline [7,12]. One explanation for this observation could be non-compliance of the general population and service providers with national malaria treatment guidelines but also could reflect non-availability of ACTs during the time of the epidemic.

Self medication was common among respondents. This involved taking antimalarials from drug shops, use of local herbs or related practices. Self treatment using drugs sourced from drug shops and general stores have been reported by many studies in malaria endemic countries. In Uganda, Ndyomugenyi [36] reported that 24.7% (n = 1627) of people who visited health facilities had practiced some form of self medication before visiting health facilities and consequently about two thirds of them reported to health facilities late (more than 24 hours after onset of illness). A similar finding in Uganda was reported by Nuwaha [37]. In a study in Southern Sudan, about half of the people were found to practice self medication before visiting a health facility three days later [38]. Self medication before seeking appropriate healthcare from health facilities have also been reported in Kenya [39] and in Tanzania [31,40]. More interestingly, a study in Nigeria reported that self medication in the form of herbal preparations is considered the first line of treatment against malaria [26]. It is evident therefore that self medication contributes to delays in seeking appropriate health care which in turn could exacerbate malaria disease in epidemic situation. Self medication can also explain the observed failure to comply with national malaria treatment guidelines which in turn affect treatment outcome and contribute to development of drug resistance.

More than half (58.7%) of respondents in Muleba district reported that their households owned at least one ITN with significantly higher ITN coverage in the non-epidemic area compared to epidemic area (66.4% vs 55.3%, p = 0.019) (Table 6). Further, in the multivariate logistic regression analysis, not using a mosquito net throughout the year was associated with an increased risk of a household being affected by malaria epidemic (Table 7). These observations points out to the possibility that low ITN coverage in the epidemic area might have contributed to increase in malaria cases during the epidemic. Apart from ITN ownership and use, knowledge of cause of malaria and household location were predictors of a household being affected by the epidemic (Table 7). The observed association between malaria epidemic and household location suggests involvement of climatic and/or environmental factors as the cause of the epidemic. This finding is supported by previous studies in Muleba [41] and other parts of East Africa [8,42,43] which observed associations between *P. falciparum *malaria epidemics and climatic factors such as increased rainfall, temperature and humidity. Likewise, the association between knowledge on malaria and malaria disease was reported by Mboera *et al *[44] in Mvomero district, Tanzania. The study observed that individuals with low knowledge on malaria experienced 2.3 times more malaria cases in their households compared to individuals with higher knowledge. The study further observed that individuals with higher knowledge on malaria were more likely to own an ITN than those with low knowledge. There were concerns by respondents about higher prices of mosquito nets to the extent of not being affordable by some community members. Higher prices of mosquito nets might have contributed to the low mosquito net coverage during the epidemic and hence less protection against malaria during the epidemic. This observation is not uncommon in Tanzania as other studies [22,45] also observed that cost in terms of money and time and any form of user fees were limiting factors for accessibility of malaria chemoprophylaxis and other health services by women and other community members. However, it was encouraging to note that majority of those who owned mosquito nets purchased the nets using their own money which indicates that communities in the study area are willing to invest in malaria control consistent with what is expected for a community with higher literacy rate and knowledge on malaria.

Despite good knowledge about malaria transmission, signs and symptoms, treatment and control, this study also revealed evidence of knowledge gaps about malaria by some respondents. Some reported that malaria is transmitted through drinking contaminated/unboiled water, staying in the sun and working in rain. In total there were 21.2% incorrect responses on malaria transmission. It is very surprising that in this study and others in malaria endemic countries, a significant proportion of respondents associated malaria with drinking contaminated water or other incorrect causes. An even higher percentage of respondents gave the same responses in a study conducted in Uganda [46] and in another similar study in Zimbabwe [32]. Similar responses were also reported in rural areas of West Africa [26,47,48]. Further, in line with two studies in West Africa [47,48], there was also a failure by most respondents in Muleba district to associate anaemia and jaundice with malaria which in turn could lead to failure to recognize malaria cases and hence failure to seek appropriate health care.

With regards to measures to prevent malaria, there were perceptions that ITNs are harmful to the health of users and more particularly to pregnant mothers. Evidence of knowledge gaps on malaria has been reported by other studies. Winch and his colleagues [49] found that people in Bagamoyo district in Tanzania failed to associate severe malaria (convulsions) in children, severe anaemia and malaria in pregnancy with malaria which in turn lead to people's failure to acknowledge the full burden and hence public health importance of the disease in the area. The knowledge gaps revealed in this study therefore indicates that some people might have opted for unsound measures of malaria control and protection and hence contributed to the increased number of malaria cases observed during the epidemic. These findings show that in order to achieve the required levels of adoption of malaria control measures, more emphasis should be placed on designing and implementation of effective health education interventions that will address knowledge gaps on malaria among communities.

## Conclusions

In conclusion, findings of this study suggest that ineffective usage of insecticide treated nets during the time of the epidemic and knowledge gaps about malaria transmission, signs and symptoms as well as prevention and control could have predisposed communities in surveyed areas to malaria epidemics. Environmental factors such as household location also played a role. These observations therefore underscore the need for improved health education among other interventions for the communities and health care providers in the district.

## Competing interests

The authors declare that they have no competing interests.

## Authors' contributions

SMK, FM, SN, GK, JM, SM, RM and LEGM designed the study. SMK and LEGM contributed to analysis and interpretation of results. SMK, FM, GK and JM supervised field data collection, SMK and CK coordinated data entry and performed data analysis, SMK drafted the manuscript, LEGM and JM revised the draft manuscript. All authors read and approved the final version of the manuscript.

## Pre-publication history

The pre-publication history for this paper can be accessed here:

http://www.biomedcentral.com/1471-2458/10/395/prepub
